# G-Quadruplex Structure in the ATP-Binding DNA Aptamer Strongly Modulates Ligand Binding Activity

**DOI:** 10.1021/acsomega.3c10386

**Published:** 2024-03-15

**Authors:** Aleah N. Edwards, Alexandria N. Iannucci, Jacob VanDenBerg, Annastiina Kesti, Tommie Rice, Srishty Sethi, Soma Dhakal, Philip M. Yangyuoru

**Affiliations:** † Northern Michigan University, 1401 Presque Isle Ave, Marquette, Michigan 49855, United States; ‡ Virginia Commonwealth University, 1001 W Main St., Richmond, Virginia 23284, United States

## Abstract

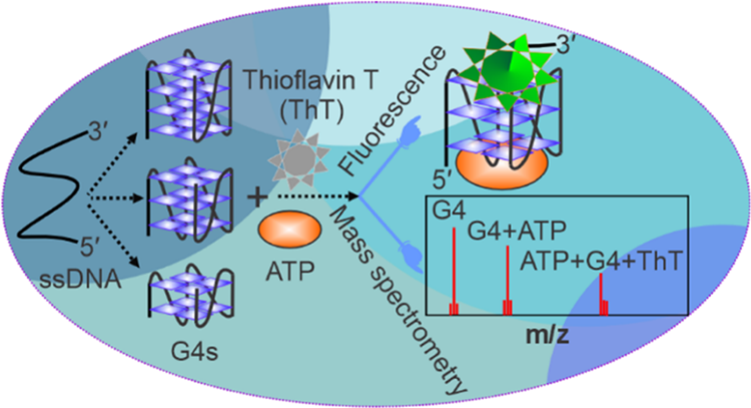

Secondary structures formed by single-stranded DNA aptamers can allow for the binding of small-molecule ligands. Some of these secondary structures are highly stable in solution and are great candidates for use in the development of molecular tools for biomarker detection, environmental monitoring, and others. In this paper, we explored adenosine triphosphate (ATP)-binding aptamers for the simultaneous detection of two small-molecule ligands: adenosine triphosphate (ATP) and thioflavin T (ThT). The aptamer can form a G-quadruplex (G4) structure with two G-quartets, and our results show that each of these quartets is equally involved in binding. Using fluorescently labeled and label-free methods, we further explored the role of the G4 motif in modulating the ligand binding property of the aptamer by making two extended variants that can form three or four G-quartet G4 structures. Through equilibrium binding and electrospray ionization mass spectrometry (ESI-MS) analysis, we observed a stronger affinity of aptamers to ATP by the variant G4 constructs relative to the native aptamer (*K*_d_ range of 0.040–0.042 μM for variants as compared to 0.15 μM for the native ATP aptamer). Additionally, we observed a dual binding of ThT and ATP to the G4 constructs in the label-free and ESI-MS analyses. These findings together suggest that the G4 motif in the ATP aptamer is a critical structural element that is required for optimum ATP binding and can be modulated for the binding of multiple ligands. These findings are instrumental for designing smart molecular tools for a wide range of applications, including biomarker monitoring and ligand binding studies.

## Introduction

Aptamer-based detection has continuously attracted a wide range of applications and research interests. Nucleic acid aptamer sequences consisting of tandem repeats of consecutive guanines tend to self-associate into four-stranded noncanonical structures called G-quadruplexes (G4s), given the proper sequence and solution conditions. G4 structures are commonly found in aptamers and are targets for various biological and chemical ligands. The ability of aptamers to adapt different structural conformations^1,2^ and the intrinsic stability of G4 structures make the G4-containing aptamers valuable for use in therapeutic and diagnostic applications. Additionally, the formation of the G4 structure can be dynamic and tunable, making it a great reporter system in biosensors and other applications. In most G4-based biosensors, binding of a target usually leads to either stabilization or disruption of the G4 structure, and the corresponding change in signal correlates with the concentration of the bound ligand. For example, the response of the sensor signal can be linked with fluorescence, where the binding of the labeled aptamer to its receptor ligands induces a conformational change on the aptamer, also called target-induced structure switching, resulting in measurable fluorescence emission.

In developing fluorometric aptamer-based sensors, fluorescence resonance energy transfer (FRET) systems are commonly used to study structural switching by labeling the oligonucleotide sequences with a fluorophore and a quencher in a molecular beacon format.^3−7^ The design of fluorescent probes for G4s can also be achieved by labeling the G4 oligonucleotide sequence with a fluorophore^8−10^ or through interaction with a fluorescent dye in close proximity by local hybridization^11−13^ or ligand.^14^ The unlabeled G4-forming sequences tend to also exhibit intrinsic fluorescence upon G4 formation compared to their unlabeled single-stranded counterparts and duplex structures.^15^ These structural and signal changes have been utilized in designing biosensors to detect a variety of analyte targets.^16,17^

Many assays have been developed for the detection of adenosine triphosphate (ATP) using the ATP-binding aptamer discovered by Huizenga and Szostak^18^ due to the biological importance of ATP and a good example of a small-molecule ligand. The ATP-binding aptamer is an example of a G4-containing aptamer with highly conserved G-rich regions that can form two G-quartets, which suggests that the active form of the aptamer is most likely a G4 structure.^18^ For the detection of ATP, both fluorescent-labeled and label-free methods have been used where the ATP-binding events are determined by an increase^8,19^ or decrease^20,21^ in fluorescence intensity upon ATP binding. Most label-free methods use thioflavin T (ThT) as a fluorescent reporter in free solution, and the ATP binding is observed as a decrease in fluorescence upon ATP-dependent displacement of ThT from the ThT-aptamer complex.

Besides fluorescence methods, another sensitive method that can be used to probe G4 structures and their interaction with small-molecule ligands is electrospray ionization mass spectrometry (ESI-MS).^22−26^ Mass spectrometry analysis of the interaction of G4s with ligands is also emerging as a powerful method for detecting G4-ligand complexes in solution and carried through the gaseous phase to unambiguously identify G4s and target ligands^27−30^ in their native conformations.

We herein used both fluorescence and mass spectrometry methods to investigate the binding interactions between ATP and DNA aptamers. Experiments were performed using the native ATP-binding aptamer capable of forming two G-quartets and two variant aptamers with extended G-tracts that can form three G-quartets and four G-quartets G4s within the oligonucleotide sequence. Interestingly, we observed strong binding of ATP to all three aptamers. While the native aptamer was originally shown to strongly bind to ATP, we found that by extending the aptamer sequence, the ATP binding activity is further enhanced. Our results show not only more robust ATP binding to the two fully formed G4 construct variants relative to the two G-quartets native ATP-binding aptamer but also two small-molecule targets; ATP and ThT simultaneously bind. These results suggest that the G4 structures modulate the ATP-binding activity.

## Experimental Section

### Materials/Chemicals

Deionized water from the Barnstead E-PURE system (Thermo Scientific) was autoclaved and filter-purified by passing through a 0.2 μm, 25 mm sterile syringe filter (Fisher) before being used to make all solutions and buffers. ATP was purchased from Thermo Scientific, and potassium chloride (KCl), sodium chloride (NaCl), and Tris Base were purchased from Fisher Scientific. EDTA was purchased from EMD Millipore Co., Billerica, Maryland. Ammonium acetate, NH_4_OAc, was purchased from Honeywell, Germany. Triethylammonium acetate, TEAAc, pH 7.0, was purchased from Sigma-Aldrich.

### Oligonucleotides

All the DNA oligonucleotides used were purchased from Integrated DNA Technologies, Coralville, IA. Some oligonucleotides were labeled with the fluorescent dye 6-carboxyfluorescein (FAM), (see Table S1). The oligonucleotides used in these experiments are listed in Table S1, three of which are guanine-rich, G4-forming oligonucleotides and two that do not form G4s as negative controls. All oligonucleotides were dissolved in TE buffer (10 mM Tris, 1 mM EDTA, pH 7.5) to about 1 mM, and the exact concentrations were determined using ultraviolet–visible (UV–Vis) absorbance at 260 nm (Cary 100 UV Spectrophotometer) and their respective molar absorptivity.

### G4 Formation

Aliquots of the dissolved oligonucleotides were added to 50 mM Tris buffer at pH 7.5 containing 100 mM KCl and heated to 95 °C for 10 min in a thermal cycler and slowly cooled to 4 °C in the presence of 100 mM KCl to form G4 structures.

### Ligand Binding Assay

To test the specificity of the sensor, we performed equilibrium ligand binding assays using ATP. Due to binding kinetics, the solutions containing varying concentrations of the ligand were incubated with the aptamer and thermal cycled in order for the G4 to form in the presence or absence of the ligand. This ensured complete binding, even at low ligand concentrations. After incubation, sample solutions were transferred from the tubes into a 96-well microplate and placed into a POLARstar Omega fluorescent plate reader to measure the fluorescence intensity.

### Data Analysis

Experiments were performed by triplicate measurements, unless stated otherwise. The fluorescence intensities with varying ligand concentrations were blank-corrected and averaged, and the normalized fraction-bound was calculated as (*F* – *F*_0_/*F*_max_ – *F*_0_), where *F*_0_ and *F* are the fluorescence intensities without ligand and with ligand at a given concentration and *F*_max_ is the fluorescence intensity at saturating concentration of ligand. The data analysis and normalization were done using Excel, and the graphs were plotted using IgorPro and fitted to single-binding site equation *Y* = *B*_max_ × *X*/(*K*_d_ + *X*) for 1:1 binding between the aptamer and ligands^18,31,32^ or *Y* = *nB*_max_ × *X*/(*K*_d_ + *X*) for two nonequivalent binding sites^4,33^ to determine which binding mode best fits our data, where *K*_d_ is the equilibrium dissociation constant or the binding affinity of the ligand for the aptamer, *B*_max_ is the maximum fraction of ligand bound, and n is the number of binding sites per aptamer construct. Maximum binding describes the point at which the binding plateaus and binding sites are saturated. Smaller dissociation constants are associated with ligands that are tightly bound to their target receptors.

### Circular Dichroism (CD) Analysis of G4 Structures

The CD spectra were recorded on a Jasco-1500 spectrometer at 25 °C in a 1 mm quartz cuvette. The spectra were collected at a scan rate of 100 nm/min and a bandwidth of 2 nm. The oligonucleotide samples for CD analysis were prepared at a concentration of 10 μM in 50 mM Tris-HCl (pH 7.5) containing 100 mM KCl. The estimated ionic concentration is 0.15 M. Samples were first thermal cycled by heating at 95 °C for 5 min and slowly cooled to room temperature to form the G4 structures. In ligand-binding experiments, the G4 structures were incubated with ATP (100 μM) for 30 min at 37 °C. The reported CD spectra are blank-corrected and smoothed using a 5-point Savitzky–Golay function.

### Gel Electrophoresis

Native polyacrylamide gel electrophoresis (PAGE) was run using thermally annealed samples in 50 mM Tris buffer at pH 7.5 containing 100 mM KCl under our standard G4 formation condition. The oligonucleotide samples (10 μM) were loaded onto a 15% gel supplemented with 50 mM KCl in 1× TBE buffer and run under 115 V for 2 h. After electrophoresis, the gel was stained in 1× SYBR Gold solution for 20 min, rinsed with nanopure water, and imaged using a Bio-Rad digital imaging station.

### HPLC-ESI-MS Analysis

ESI mass spectra were obtained using a Shimadzu LCMS-8040 equipped with a triple quadrupole mass analyzer. The stationary phase used is a C_18_ column with high-performance liquid chromatography (HPLC) grade water and 50 mM TEAAc (pH 7.0) buffer as the mobile phases. All spectra were obtained in negative ion mode. Native electrospray mass spectra were obtained with the following settings: a Q3 scan range of 500–2000 *m*/*z*, scan speed of 1578 μ/s, ESI interface nebulizing gas flow of 3 L/min, DL temp of 250 °C, drying gas flow of 15 L/min, ESI interface voltage of 0 kV, PG Vacuum of 1.0 E^2^, and CID Gas of 230 kPa. The G4 samples used were either 5 or 10 μM with sample injection volumes of 10 μL. The G4 structures in the aptamer sequences were annealed with ATP under the required high KCl condition (50 mM KCl, 50 mM Tris, pH 7.5) at a molar concentration of 1 G4/5 ATP. Aliquots were subsequently taken and diluted with TEAAc buffer, pH 6.8 down to 1 mM KCl, 1 mM Tris, and 100 mM TEAAc final reaction buffer concentration. The estimated ionic concentration is 0.12 M. The reaction mix was transferred into an autosampler vial with an insert and run on Shimadzu HPLC-MS.

## Results and Discussion

### CD Analysis of Aptamer Sequences

**Figure 1:**
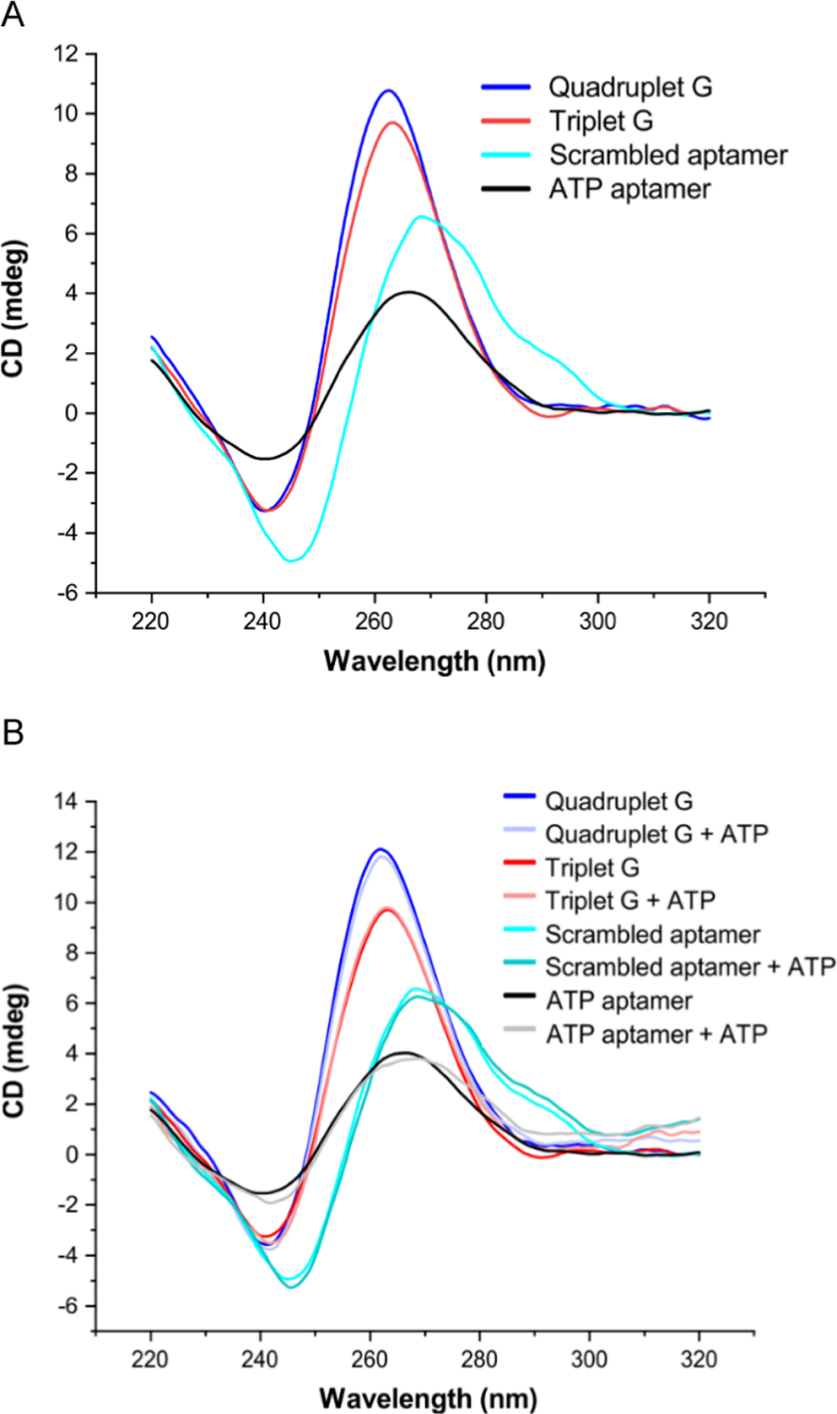
Structural analysis of aptamer sequences. (A) Circular dichroism spectroscopic analysis of the structural conformation of the aptamer and scrambled oligonucleotides in solution. (B) Comparison of circular dichroism spectra of aptamer and scrambled oligonucleotide sequences in the absence and presence of ATP.

The ATP-binding DNA aptamer was selected against ATP by affinity chromatography^18^ and structural analysis by NMR.^33^ The proposed active structure of the ATP aptamer consists of a stack of two G-quartets, two short stems, and two conserved adenosine residues.^18^ The involvement of the G4 structure in ligand binding was investigated by systematically increasing the number of guanines (increased to 3 and then 4 guanines per G-rich stretch) in the aptamer sequence to determine whether they form the full three G-quartets and four G-quartets G4s and how that will impact the ATP binding pocket of the aptamer and its binding ability. We first performed CD experiments to determine the structural conformations of the oligo sequences in the solution. The CD spectra of the structures formed by the ATP aptamer and the two extended potential G4-forming sequence variants show peak maxima at 260 nm and minima at 240 nm. Such CD signatures are characteristic of parallel G4 conformations.^34,35^ On the other hand, the scrambled ATP aptamer, which does not have consecutive guanines, shows a peak at ∼275 nm depicting a random coil^36,37^ structure (Figure 1A). These CD results indicate the formation of parallel G4 structures by the native ATP aptamer and the two variants: triplet G and quadruplet G oligonucleotides in solution and no G4 structure in the scrambled aptamer sequence.

We next probed whether the conformations of the G4 structures formed in solution are perturbed upon ATP binding. This was done by thermal cycling each oligonucleotide in 50 mM Tris buffer, pH 7.5, and 100 mM KCl to form the G4 structures followed by incubating with 10-fold ATP ligand at 37 °C. We observed no structural changes in the CD profiles after incubating with the ATP ligand, which suggests that the binding of small-molecule ATP does not perturb G4 CD signatures (Figure 1B).

We next examined whether the oligonucleotides for the G4s and control constructs formed alternative structures or aggregates instead of G4s by running native polyacrylamide gel electrophoresis (PAGE) (Figure S1). As expected, no obvious aggregates were observed in the triplet G and quadruplet G G4 constructs and in the control oligonucleotides (GAT and scrambled). There are two slower moving bands that may be alternative structures formed by the aptamers. However, the G4 bands are the major ones and the two slower bands account for less than 20% of the aptamer G4 band at the bottom.

### Label-Free Fluorescence Light-Up Method for Detecting G4 Structures

**Figure 2:**
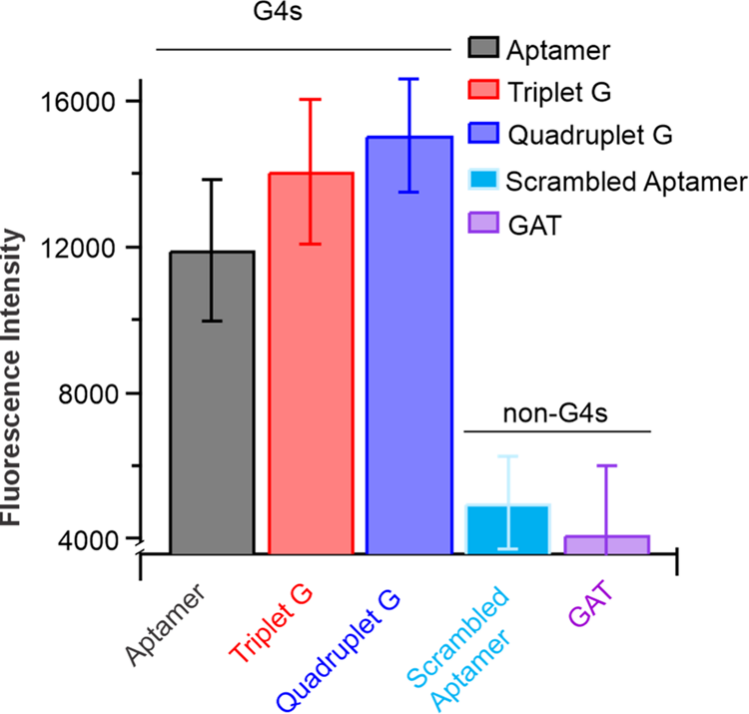
Thioflavin T (ThT) specifically binds to G4 structures, leading to an increase in the fluorescence intensity of ThT in a fluorescence light-up experiment.

To further support the results of the G4 structures found in the CD analysis, we next used a label-free fluorescence method for detecting the G4 structures. Thioflavin T (ThT) is a well-known fluorescent dye that selectively targets G4 structures and certain amyloid proteins.^38^ ThT dye has been shown to specifically target G4 structures resulting in a large increase in ThT fluorescence emission in free solution.^12,39^ The potential G4-forming and control sequences were incubated with ThT to further probe the structures formed by the aptamer and the variant aptamer sequences in solution. We observed about a 3- to 4-fold increase in fluorescence intensity upon incubating the native ATP aptamer and the two elongated variants with ThT relative to the control sequences that do not form G4 structures (Figure 2). These results indicate that the ATP binding aptamer and the two variant constructs indeed form G4 structures, which are specifically targeted by the ThT in solution.

### ATP Ligand and ThT Both Bind to the G4 Constructs under Slow Annealing Condition

**Figure 3:**
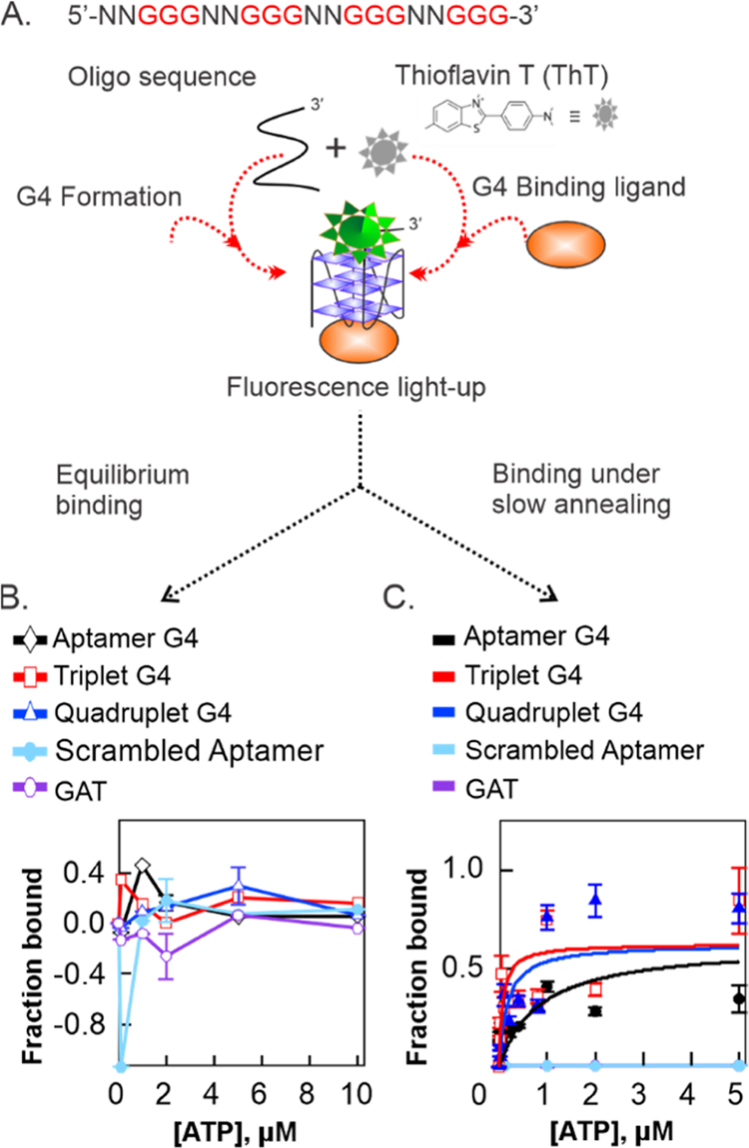
Analysis of ATP binding to the ATP aptamer and variant aptamer G4 structures. (A) Schematic of the G4-based ATP binding method indicating fluorescence light-up upon G4 formation by the oligonucleotides and ThT binding. (B) Equilibrium binding curves at varying ATP concentrations and (C) under slow annealing conditions. The binding curves were fitted with the single-binding site equation to obtain the following *K*_d_ values: *K*_d_ (aptamer) = 0.83 μM, *K*_d_ (quadruplet G) = 0.17 μM, and *K*_d_ (triplet G) = 0.071 μM.

After determining that ThT specifically targets G4 structures (consistent with the literature^12,39^), we next designed experiments exploring the ThT-G4 interactions to probe the binding properties of ATP and the ATP-binding aptamer and aptamer variants. Equilibrium binding assay was performed by first forming the G4 structures and then subsequently incubating with a fixed concentration of ThT (0.5 μM) and varying concentrations of ATP for 1 h at 37 °C in a water bath (Figure 3A). The hypothesis was that if ATP binding directly competes with ThT, it would displace ThT from the G4 structure resulting in a decrease in the fluorescence. However, if ATP and ThT bind cooperatively, the fluorescence will remain either unchanged or increase depending on the interaction. Indeed, we observed no change in fluorescence intensity with increasing ATP concentration under equilibrium conditions, suggesting no detectable ATP binding activity up to 10 μM ATP (Figure 3B). There was no detectable difference in the fluorescence intensity between the G4 structures, the scrambled aptamer, and the GAT negative controls, which cannot form G4 structures. These results indicate a competitive displacement of ThT by the ATP ligand. However, when the G4 structure was incubated with ThT and ATP in solution followed by thermal cycling and slow annealing for 1 h, we observed an increase in fluorescence intensity with an increased concentration of ATP. These results demonstrated a robust binding between ATP and the G4 constructs. This observation was further validated by no activity toward the scrambled aptamer and GAT negative control constructs that are incapable of forming G4 structures (Figure 3C). These results suggest that under these experimental conditions, the ATP ligand did not displace the ThT dye, but both ligands bind simultaneously. These data were best fitted to the single-site binding model and is consistent with previous reports.^40,41^ Interestingly, the binding affinity is stronger for the two extended aptamer variants resulting in *K*_d_ values of 0.83, 0.071, and 0.17 μM for the native aptamer, triplet G, and quadruplet G, respectively. The measured *K*_d_ values yielded 12-fold and 5-fold increase in binding affinity for the triplet G and quadruplet G, respectively, relative to the native aptamer, indicating a higher ATP affinity for the extended variant aptamers.

### Fluorescently Labeled Method for Determining ATP Binding Affinity

**Figure 4:**
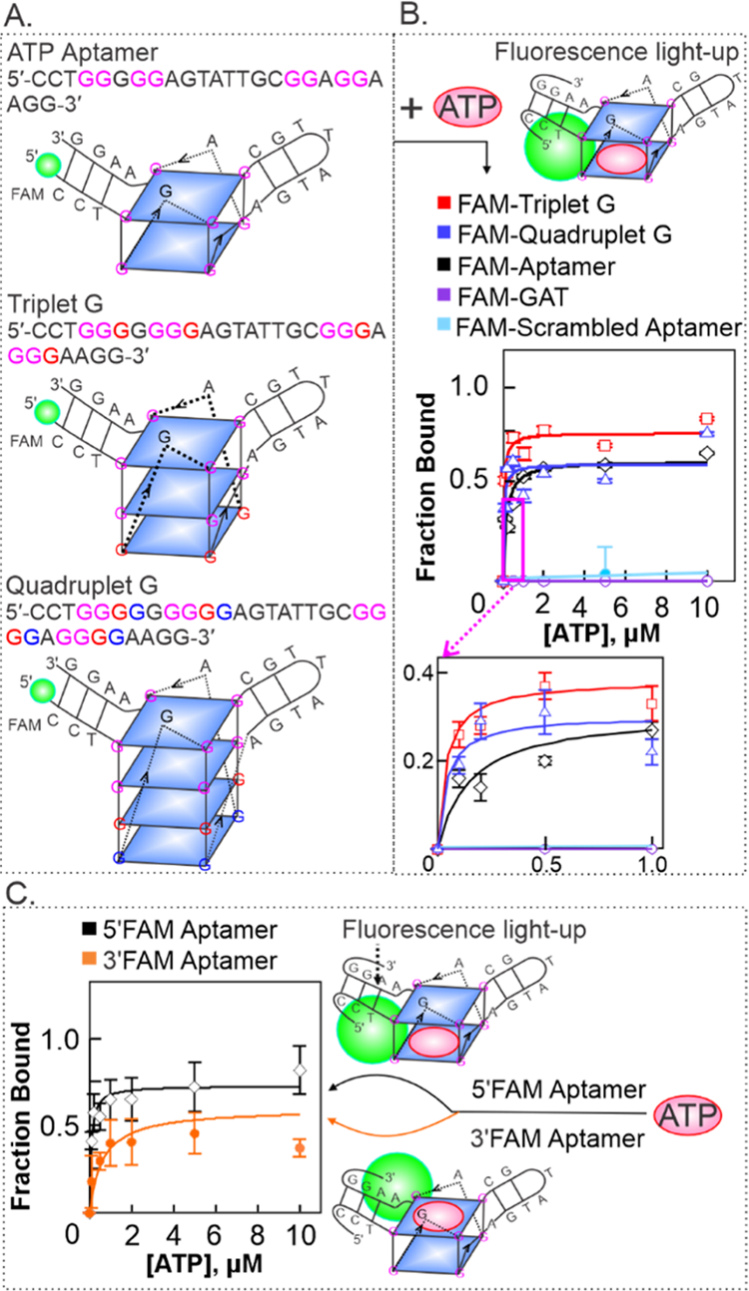
Fluorescently labeled method for determining ATP binding activity using FAM-labeled oligonucleotides. (A) Sequences of the G4-forming oligonucleotides and their respective G4 structures, (B) ATP binding curves for the various G4 constructs and scrambled aptamer and GAT negative control sequences, and (C) binding curves for the ATP-binding aptamer labeled with FAM fluorophore at the 5′-end (top) or 3′-end (bottom). The *K*_d_ for the 5′-FAM aptamer is 0.15 μM and for the 3′-FAM aptamer is 0.62 μM.

The results showing the formation of G4 structures by the ATP-binding aptamers and dual binding of ATP and ThT to these G4 structures in a label-free format led us to further probe this binding process with a fluorescently labeled approach. This was done by directly labeling the G4-forming oligonucleotides with a fluorophore. By this format, we can directly determine whether ATP binding will result in fluorescence light-up, as observed with the label-free ThT fluorophore in solution. For these experiments, the ATP aptamer oligonucleotides and the variant ATP aptamers were covalently labeled with the carboxyfluorescein dye (FAM) at the 5′-end (IDT DNA) (Figure 4A and Table S1). The labeled constructs (0.50 μM) were slowly annealed with varying concentrations of ATP (0–10 μM), and fluorescence was measured. It was expected that upon ATP binding, the G4-ATP complex would be in close proximity to the fluorophore, resulting in an increase in the fluorescence intensity. Indeed, we observed an increase in fluorescence intensity with increased ATP concentration (Figure 4B). The increasing fluorescence intensity corresponds to the amount (fraction) of ATP bound to the aptamer. Interestingly, the aptamer and the variant constructs: triplet G and quadruplet G, all demonstrated robust binding affinity to ATP with a *K*_d_ value of 0.15 μM for the native aptamer, 0.040 μM for triplet G, and 0.042 μM for quadruplet G (Figure 4B, middle and inset). The strong binding between ATP and the aptamer observed here is consistent with earlier reports.^9,42^ However, and most interestingly, we observed more robust binding between ATP and the variant aptamers than the native ATP-binding aptamer. These *K*_d_ values show ∼4-fold stronger binding affinity for ATP by the variant aptamer constructs relative to the native aptamer. Compared to the label-free method (Figure 3), the fluorescently labeled method produced lower *K*_d_ values in general for the native and variant aptamers indicating a stronger binding affinity with *K*_d_ aptamer_label-free_/*K*_d_ aptamer_labeled_ = 6-fold, *K*_d_ triplet *G*_label-free_/*K*_d_ triplet *G*_labeled_ = 2-fold, and *K*_d_ quadruplet *G*_label-free_/*K*_d_ quadruplet *G*_labeled_ = 4-fold. The higher *K*_d_ values obtained in the label-free method are probably the indication of competitive displacement of the ATP ligand by the freely diffusing and more labile ThT molecules. The competitive displacement or inhibition is however eliminated in the fluorescently labeled method where the fluorophore is attached to a fixed position on the G4 structure and not freely moving to compete with the ATP molecules for the same binding site. Taking together, these results demonstrate that the G4 motif in the aptamer and variant sequences modulates the ATP-binding activity. The results obtained using the fluorescently labeled approach showed much stronger binding between ATP and aptamers, which indicates that the fluorescently labeled approach is a robust technique for determining the ATP binding activity using G4-forming aptamers.

### Probing the Contribution of G-Quartets in Ligand Binding

With the finding that the fluorescently labeled method is more sensitive, we next asked whether the labeling position would affect the fluorescence signal and/or the ATP binding activity. To achieve this, we used the native ATP-binding aptamer and designed experiments to determine the contribution of each G-quartet to ligand binding by systematically labeling the 5′- and 3′-ends of two separate native ATP-binding aptamers with 6-carboxyfluorescein (FAM). The 5′-end label would bring the fluorophore closer to the top G-quartet, while the 3′-labeled fluorophore is closer to the bottom G-quartet (shown in the cartoon in Figure 4C, right). This design enabled us to directly monitor the contribution of each G-quartet to ligand binding by performing equilibrium binding assays. Under varying ATP concentrations, we observed low dissociation constants, *K*_d_ values, indicating strong binding for both 5′- and 3′- labeled aptamer albeit 4-fold lower *K*_d_ value in the 5′- FAM aptamer (Figure 4C). These results suggest that both G-quartets in the ATP-binding aptamer are associated with the binding site of the aptamer and contribute to binding. These results also indicate that the position of the fluorescent label did not impact the G4 formation and the binding pocket of the ATP aptamer but enabled measuring the contribution of the G4 motif in the binding of ATP.

### HPLC-ESI-MS Analysis of ATP Binding of the G4 Structures

**Figure 5:**
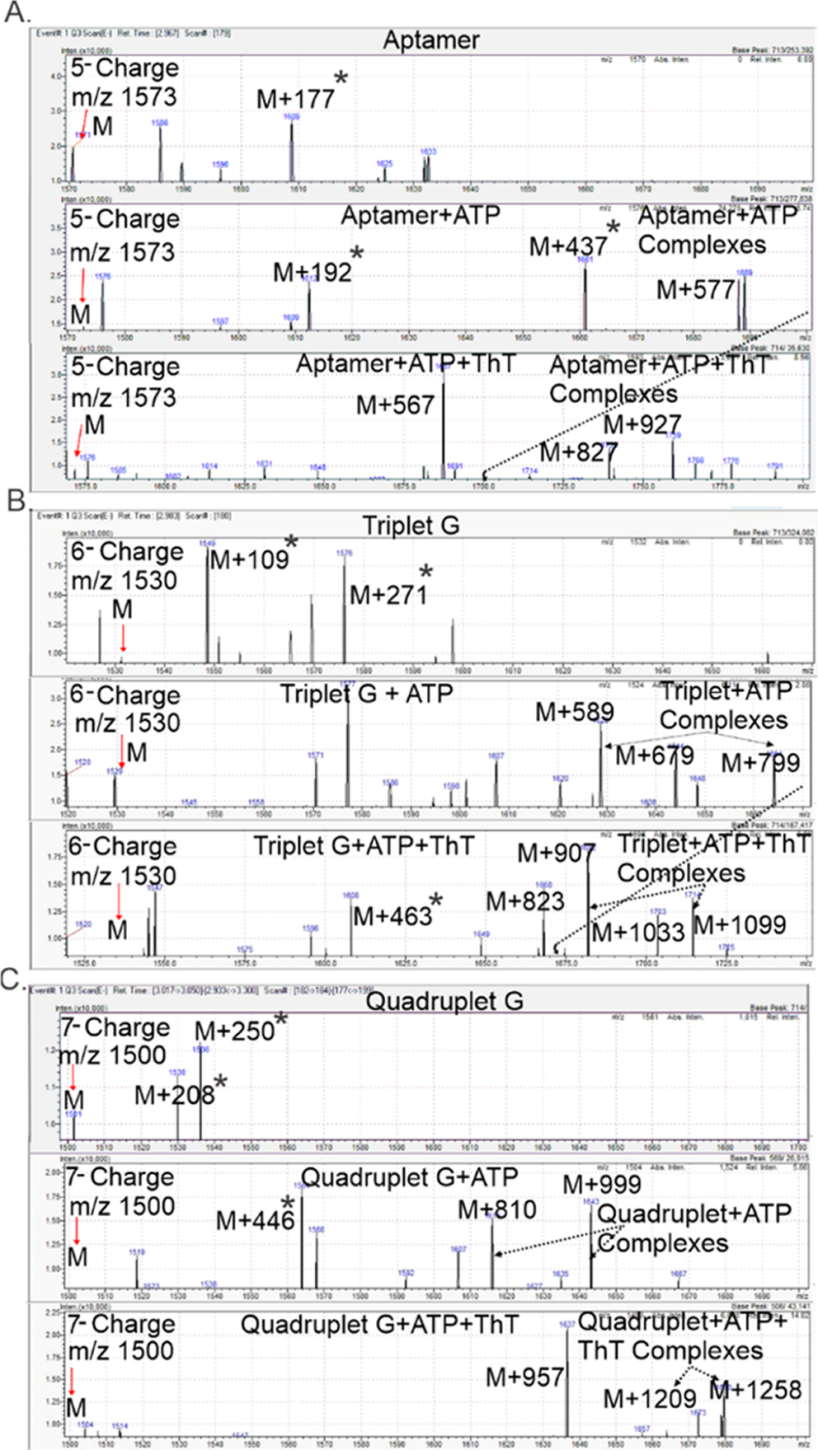
ESI-MS analysis of the G4 constructs alone or in the presence of ATP and ThT ligands: (A) aptamer alone (top) and with ATP aptamer + ATP (middle) and aptamer + ATP + ThT (bottom); (B) triplet G alone (top) and with ATP (middle) and triplet G + ATP + ThT (bottom); and (C) quadruplet G alone (top) and in the presence of ATP (middle) and quadruplet G + ATP + ThT (bottom). Parent molecular ion mass for each construct is indicated as M at the corresponding *m*/*z* and the bound G4-ATP complexes are indicated as ≥M + 507 peaks, while the G4 + ATP + ThT complexes are indicated as ≥M + 826 peaks. The identification of the peaks with asterisks is provided in the Supporting Material.

We next asked if the binding between the G4 constructs and the ATP ligand in solution is stable enough to survive the electrospray ionization and be detected in the gas phase using ESI mass spectrometry. To do this, the G4 constructs were annealed with ATP in the molar ratio of 1 G4/5 ATP in a solution containing 50 mM KCl and 50 mM Tris at pH 7.5. Before mass spectrometry analysis, the solution was diluted down to 1 mM KCl, 1 mM Tris, and 100 mM triethylammonium acetate (TEAAc) to replace the hard-to-ionize KCl salt with the more volatile TEAAc while still maintaining the ionic strength and G4 structures as was demonstrated previously.^27^ ESI-MS experiments were run in the negative ion mode, and the dominant charge states were identified. The neutral masses of the detected species were calculated to enable the unambiguous identification of the G4-ligand complexes formed in solution and carried into the gas phase and to the mass analyzer. We observed various charge states for the G4 structures with 5^–^, 6^–^, and 7^–^ being the most dominant charge states for the aptamer, triplet G, and quadruplet G constructs, respectively. The mass spectra for the three G4 constructs alone show peaks for the dominant charge states, with M being the calculated parent molecular ion peaks at the indicated *m*/*z* values (Figure 5A–C, top). The spectra obtained upon incubating the aptamer alone with the ATP ligand (1 aptamer/5 ATP ratio) show peaks corresponding to the Aptamer + ATP complexes at M + 577 with the molecular weight of ATP given as 507 g/mol (Figure 5A, middle). The triplet G construct shows three dominant peaks at M + 589, M + 679, and M + 799 corresponding to the triplet G + ATP complexes (Figure 5B, middle). The quadruplet G construct shows two dominant peaks at M + 810 and M + 999 corresponding to the quadruplet G + ATP complexes (Figure 5C, middle). These complexes unambiguously represent G4s-ATP complexes of each G4 construct, further supporting our fluorescence results that there is strong binding between the three G4 constructs and ATP ligand.

### HPLC-ESI-MS Analysis Shows Dual Binding of ATP and ThT Ligands to G4 Structures

To probe if the ATP and ThT ligands can both bind to the same G4 construct, the G4 constructs were slow-annealed with the ATP and ThT ligands in solution (in 1 G4/5 ATP/5 ThT ratio). Similar to the results of the ATP alone experiments, peaks corresponding to the G4 + ATP + ThT complexes were observed, indicating dual binding of both ligands to the G4 structures in solution (Figure 5A–C, bottom). The aptamer construct shows dominant peaks at a higher *m*/*z* range which are clearly distinct from the Aptamer + ATP complex peaks observed. The molecular weight of ATP + ThT is 826 and the Aptamer + ATP + ThT complexes are expected to be ≥M + 826. Indeed, we observed peaks corresponding to M + 827 and M + 927, which can be ascribed as Aptamer + ATP + ThT complex peaks (Figure 5A, bottom). For the triplet G construct, upon incubation with the two small-molecule ligands, the dominant peaks were also observed at higher *m*/*z* values beyond the ATP-bound complexes and at the ≥M + 826 if both ligands are bound. The triplet G + ATP + ThT complexes observed are indicated as M + 907, M + 1033, and M + 1099 (Figure 5B, bottom). A similar result was obtained for the quadruplet G construct with the quadruplet G + ATP + ThT complexes observed as M + 1209 and M + 1258 (Figure 5C bottom). The peaks with asterisks denote other dominant complexes of the G4s with the buffer components that are not the ATP- and ThT-G4 complexes. The plausible analysis and identification of these peaks are provided in the supporting material (Figure S2).

Previous work by Sara Richter’s group (DOI: 10.1021/acs.analchem.7b0128) suggested that even at low K^+^ concentration (<1 mM), the K^+^ ions are still able to coordinate between quartets in the G4. Indeed, looking at our mass spectrometry data, we do observe one K^+^ ion coordinating the quartets in the aptamer, 2 K^+^ ions in the triplet G, and 2 K^+^ and 3 K^+^ ions in the quadruplet G4 (see Supporting Material). These results corroborate well with the previous finding that the K^+^ ions coordinate between quartets.

Taking together, these complexes unambiguously demonstrate the dual binding of the two small molecules ligands: ATP and ThT to the three G4 constructs tested.

## Conclusions

In aptamer-based detection, secondary structures formed by the single-stranded DNA aptamers allow for the binding of small-molecule ligands, and they can be used to screen potential drug candidates as binding partners. One such aptamer is the ATP-binding aptamer, which has been shown to fold into a secondary structure including a hairpin and a G4 motif consisting of only two G-quartets. We investigated the contribution of the two G-quartets in ATP binding and found that both G-quartets are equally involved, albeit slightly stronger, binding to the top (5′-end G-quartet). We then modified the native ATP-binding aptamer by extending its sequence to enable the formation of fully formed three- and four-G-quartet G4 structures and explored the role of the G4 motifs in modulating the ligand binding activity. Our results demonstrate that a fully formed G4 structure in the ATP-binding aptamer sequence enhanced the binding ability of the aptamer by ∼4-fold. Our results, using both ESI mass spectrometry and fluorescence-based methods, show that the variant aptamer constructs bind ATP with a higher affinity relative to the native aptamer. In addition, the two small-molecule ligands, ATP and ThT, can simultaneously bind to these G4-containing aptamers. These results demonstrate that the G4 motif in the ATP-binding aptamer can be modulated to improve its target binding activity. These findings are instrumental for designing G4-based smart molecular tools for a wide range of applications including biomarker monitoring and ligand binding studies.

## Supporting Information Available

Oligonucleotide sequences used in the experiments (Table S1); polyacrylamide gel electrophoresis (PAGE) analysis of G4 and control constructs; potassium ions coordination of G-quartets; peaks’ identification of LCMS data (PDF)
