# Supplementary material for: G-Quadruplex Structure in the ATP-Binding DNA Aptamer Strongly Modulates Ligand Binding Activity
Source: ACS Omega. 2024 Mar 15;9(12):14343–50. doi: 10.1021/acsomega.3c10386 (PMC10976393; doi:10.1021/acsomega.3c10386)
Supplement: Supplementary file 1 — ao3c10386_si_001.pdf [file ao3c10386_si_001.pdf]

## Supporting Information

# The G-quadruplex structure in the ATP-binding DNA Aptamer strongly modulates ligand binding activity

Aleah N. Edwards[#‡], Alexandria N. Iannucci[#‡], Jacob VanDenBerg[#], Annastiina Kesti[#], Tommie Rice[#], Srishty Sethi[$], Soma Dhakal[$], Philip M. Yangyuoru[#]*

[#]Northern Michigan University, 1401 Presque Isle Ave, Marquette, Michigan 49855

[$]Virginia Commonwealth University, 1001 W Main St., Richmond, Virginia 23284

### Table S1. Oligonucleotide sequences used in experiments

| | |
|---|---|
| ATP Aptamer | 5′-CCTGGGGGAGTATTGCGGAGGAAGG-3′ |
| Triplet G | 5′-CCTGGGGGGGAGTATTGCGGGAGGGAAGG-3′ |
| Quadruplet G | 5′-CCTGGGGGGGGGAGTATTGCGGGGAGGGGAAGG-3′ |
| Scrambled Aptamer | 5′-GTGCGCGAGTGTGAGCGAGAGTGAG-3′ |
| GAT | 5′-GATAGCGTCACTCGATCT-3′ |
| Labeled oligonucleotides | |
| FAM-ATP Aptamer | 5′-FAM-TTCCTGGGGGAGTATTGCGGAGGAAGG-3′ |
| ATP Aptamer-FAM | 5′-TTCCTGGGGGAGTATTGCGGAGGAAGG-FAM-3′ |
| FAM-Triplet G | 5′-FAM-TTCCTGGGGGGGAGTATTGCGGGAGGGAAGG-3′ |
| FAM-Quadruplet G | 5′-FAM-TTCCTGGGGGGGGGAGTATTGCGGGGAGGGGAAGG-3′ |
| FAM-Scrambled Aptamer | 5′-FAM-TTGTGCGCGAGTGTGAGCGAGAGTGAG-3′ |
| FAM-GAT | 5′-FAM-TTGATAGCGTCACTCGATCT-3′ |

**<u>Polyacrylamide Gel Electrophoresis (PAGE) Analysis of G4 and Control Constructs</u>**

We ran a native polyacrylamide gel electrophoresis (PAGE) for high resolution separation. Below is the gel image obtained. The lanes are marked for the samples. The gel was run using thermally annealed samples in 50 mM Tris buffer at pH 7.5 containing 100 mM KCl and heated to 95 °C for 10 min in a thermal cycler and slowly cooled to 4 °C. The oligonucleotide samples (10 µM) were loaded onto a 15 % PAGE supplemented with 50 mM KCl in 1× TBE buffer and ran under 115 V for 2 hrs. After electrophoresis, the gel was stained in 1× SYBR Gold solution for 20 mins, rinsed with DI water and imaged using a Bio-Rad digital imaging station.

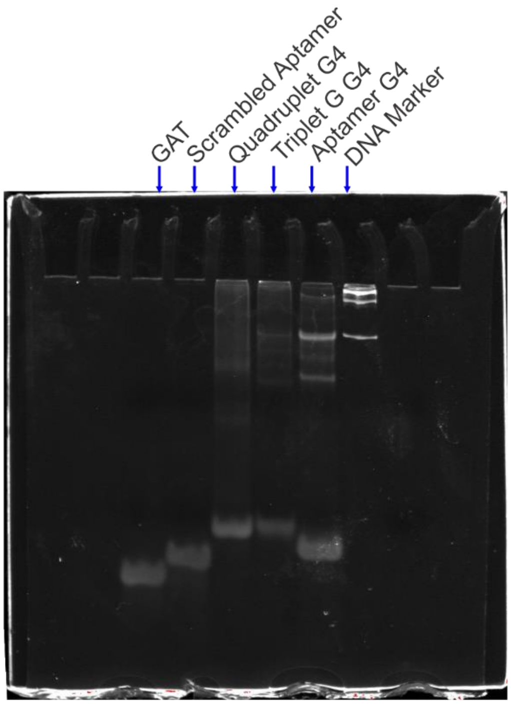


**Figure S1**. Native PAGE analysis of G4s and control constructs.

As expected, no obvious aggregates are observed in the Triplet G and Quadruplet G G4 constructs and in the control oligonucleotides (GAT and Scrambled). There are however, two slower moving bands for the aptamer which may be alternative structures formed by aptamer. The two slower bands account for less than 20% of the Aptamer G4 band at the bottom.

## Peak Identification/Assignment

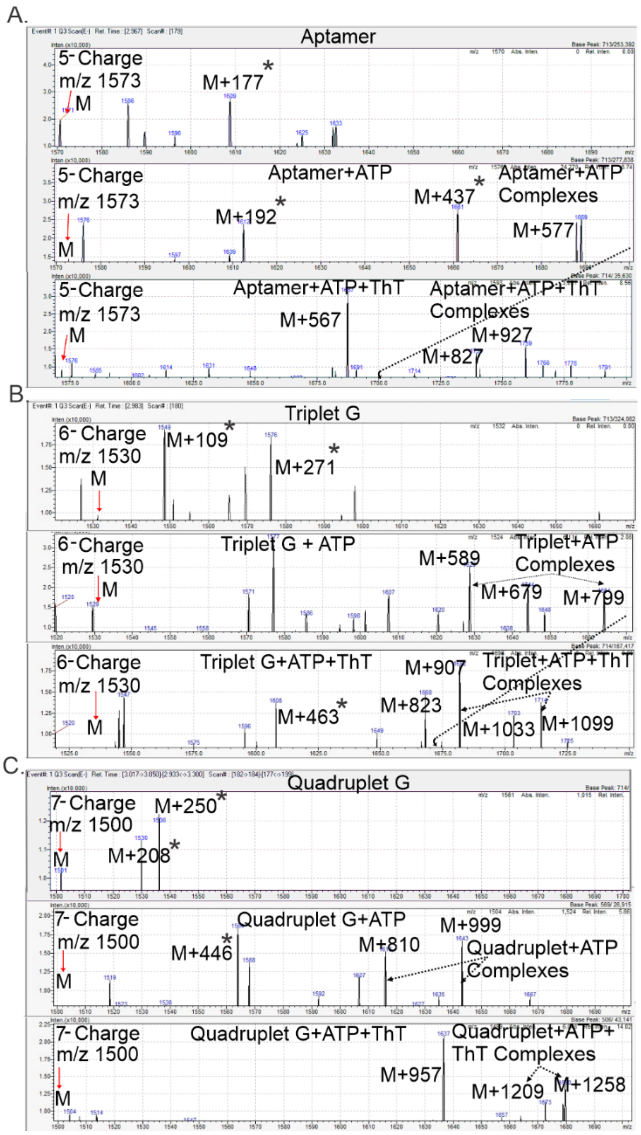


**Figure S2**. ESI-MS analysis of the G4 constructs

Plausible identification of the peaks with asterisks in the aptamer, triplet G, and quadruplet G chromatograms is provided below:

Aptamer:

M+177 peak = $[\text{aptamer}+3\text{OAc}]^{5-}$
M+192 peak = $[\text{Aptamer}+\text{TEAAc}+\text{K}^{+}-8\text{H}^{+}]^{5-}$
M+437 = $[\text{Aptamer}+4\text{TE}+\text{K}^{+}-6\text{H}^{+}]^{5-}$

Triplet:
M+109 = $[\text{Triplet}+2\text{OAc}-9\text{H}^{+}]^{6-}$
M+271 = $[\text{Triplet}+2\text{TEA}+2\text{K}^{+}-9\text{H}^{+}]^{6-}$
Trip +ATP+ThT: M+463 = $[\text{Triplet}+4\text{TEA}+\text{OAc}]^{6-}$

Quadruplet:
M+208 = [Quad+3OAc+$K^+$-8$H^+$]$^{7-}$
M+250 = [Quad+3OAc+2$K^+$-5$H^+$]$^{7-}$
Quadruplet + ATP: M+446 = [Quad+2TEAAc+3$K^+$]$^{7-}$

**<u>Potassium ions Coordination of G-quartets</u>**

The presence of $K^+$ was not determined using dedicated experiments. Previous work by Sara Richter's group (DOI: 10.1021/acs.analchem.7b0128) suggested that even at a low $K^+$ concentration (< 1 mM), the $K^+$ ions are still able to coordinate between quartets in the G4. Indeed, looking at our mass spectrometry data, we do observe one $K^+$ ion coordinating the quartets in the aptamer, 2 $K^+$ ions in the triplet G and 2 $K^+$ and 3$K^+$ ions in the quadruplet G4 as shown below. These results corroborate well with the previous finding that the K+ ions coordinate between quartets.

Aptamer
M+192 peak = [Aptamer+TEAAc+$K^+$-8$H^+$]$^{5-}$
M+437 = [Aptamer+4TE+$K^+$-6$H^+$]$^{5-}$

Triplet
M+271 = [Triplet+2TEA+2$K^+$-9$H^+$]$^{6-}$

Quadruplet
M+208 = [Quadruplet+3OAc+$K^+$-8$H^+$]$^{7-}$
M+250 = [Quadruplet+3OAc+2$K^+$-5$H^+$]$^{7-}$
Quad + ATP: M+446 = [Quadruplet+2TEAAc+3$K^+$]$^{7-}$
